# Spinal cord injury reprograms muscle fibroadipogenic progenitors to form heterotopic bones within muscles

**DOI:** 10.1038/s41413-022-00188-y

**Published:** 2022-02-25

**Authors:** Hsu-Wen Tseng, Dorothée Girard, Kylie A. Alexander, Susan M. Millard, Frédéric Torossian, Adrienne Anginot, Whitney Fleming, Jules Gueguen, Marie-Emmanuelle Goriot, Denis Clay, Beulah Jose, Bianca Nowlan, Allison R. Pettit, Marjorie Salga, François Genêt, Marie-Caroline Le Bousse-Kerdilès, Sébastien Banzet, Jean-Pierre Lévesque

**Affiliations:** 1 Mater Research Institute—The University of Queensland, Woolloongabba, QLD Australia; 2 Institut de Recherche Biomédicale des Armées (IRBA), INSERM UMRS-MD, 1197 Clamart, France; 3 INSERM UMRS-MD 1197, Université de Paris-Saclay, Hôpital Paul Brousse, Villejuif, France; 4 INSERM UMS-44, Université de Paris-Saclay, Hôpital Paul Brousse, Villejuif, France; 5 UPOH (Unité Péri Opératoire du Handicap, Perioperative Disability Unit), Physical and Rehabilitation Medicine department, Raymond-Poincaré Hospital, Assistance Publique – Hôpitaux de Paris (AP-HP), Garches, France; 6 Université de Versailles Saint Quentin en Yvelines, UFR Simone Veil - Santé, END:ICAP INSERM U1179, Montigny le Bretonneux, France

**Keywords:** Bone, Pathogenesis

## Abstract

The cells of origin of neurogenic heterotopic ossifications (NHOs), which develop frequently in the periarticular muscles following spinal cord injuries (SCIs) and traumatic brain injuries, remain unclear because skeletal muscle harbors two progenitor cell populations: satellite cells (SCs), which are myogenic, and fibroadipogenic progenitors (FAPs), which are mesenchymal. Lineage-tracing experiments using the Cre recombinase/LoxP system were performed in two mouse strains with the fluorescent protein ZsGreen specifically expressed in either SCs or FAPs in skeletal muscles under the control of the *Pax7* or *Prrx1* gene promoter, respectively. These experiments demonstrate that following muscle injury, SCI causes the upregulation of PDGFRα expression on FAPs but not SCs and the failure of SCs to regenerate myofibers in the injured muscle, with reduced apoptosis and continued proliferation of muscle resident FAPs enabling their osteogenic differentiation into NHOs. No cells expressing ZsGreen under the *Prrx1* promoter were detected in the blood after injury, suggesting that the cells of origin of NHOs are locally derived from the injured muscle. We validated these findings using human NHO biopsies. PDGFRα^+^ mesenchymal cells isolated from the muscle surrounding NHO biopsies could develop ectopic human bones when transplanted into immunocompromised mice, whereas CD56^+^ myogenic cells had a much lower potential. Therefore, NHO is a pathology of the injured muscle in which SCI reprograms FAPs to undergo uncontrolled proliferation and differentiation into osteoblasts.

## Introduction

Neurogenic heterotopic ossifications (NHOs) are pathological heterotopic bones that develop in peri-articular muscles^1,2^ following severe lesions of the central nervous system (CNS), such as spinal cord injuries (SCIs), traumatic brain injuries, strokes and cerebral anoxia. NHOs are frequent, with an incidence of 10%–23% in patients with traumatic brain injury and 10%–53% in patients with SCIs, increasing to 68% in victims of severe combat blast injuries involving the spine.^3–8^ NHOs develop most frequently at the hip, elbow, knee and shoulder.^2,9^ Because of their large size, NHOs are highly incapacitating, causing substantial pain and a gradual reduction in the range of motion of affected limbs, often progressing to complete ankylosis.^2,9^ This process exacerbates functional disabilities by increasing the difficulty in sitting, eating and dressing.^10^ NHO growth can also cause nerve and blood vessel compression, further increasing patient morbidity.^11,12^ Although this pathology has been known for just over 100 years, treatment is currently limited to surgical resection after NHOs have matured,^2,10,13,14^ a procedure that is challenging, particularly when ossifications entrap the whole joint and adjacent large blood vessels and nerves. The development of improved treatments for NHOs has been slow, and trials of pharmacological interventions have continued to show limited effectiveness,^15,16^ reflecting the current limited knowledge on the etiology, pathogenesis and pathobiology of NHOs. Indeed, the initial causal mechanisms that trigger NHOs are unique to this pathology, as they involve severe CNS trauma^17,18^ rather than other forms of trauma, such as body burns, or activating mutations of osteogenic genes, such as bone morphogenetic protein type I receptor *ACVR1*, in fibrodysplasia ossificans progressiva (FOP).^17^ Therefore, it is essential to uncover the mechanisms of NHO pathogenesis to identify potential therapeutic targets and treatments that will reduce NHO development and remove the need for very invasive and delicate surgical resections.^17^

Our group previously reported the first mouse model of NHOs in which NHOs spontaneously develop when an SCI is combined with muscle injury without additional nonphysiological manipulations, such as the overexpression of bone morphogenetic protein (BMP) transgenes or the insertion of hyperactive mutants of BMP receptors.^19^ Our model revealed that SCI causes a further exacerbation of the inflammatory response in injured muscles with exaggerated Ly6C^bright^ inflammatory monocyte/macrophage infiltration and persistent accumulation of the inflammatory cytokine oncostatin M (OSM), leading to persistent activation of JAK1/2 tyrosine kinases and signal transducer and activator of transduction-3 (STAT3), which in turn promote NHOs instead of muscle repair.^19–21^

How injured skeletal muscles generate heterotopic bones instead of regenerating functional myofibers following severe lesions of the CNS remains a fascinating stem cell biology question and may reveal novel therapeutic strategies to treat NHOs.^17,18^ Adult skeletal muscles contain two populations of stem/progenitor cells: (1) satellite cells (SCs), residing within the myofiber under the myofiber basal lamina, which regenerate myoblasts and myocytes following injury and are as such true muscle stem cells,^22–24^ and (2) fibroadipogenic progenitors (FAPs) residing in the interstitial space between myofibers.^25^ Unlike SCs, FAPs are of mesenchymal origin and do not regenerate myoblasts.^25^ Muscle repair following injury is a highly orchestrated process that involves the coordinated recruitment of both SCs and FAPs as well as macrophages. Upon muscle injury, FAPs proliferate transiently for the first 3 days in mice and then undergo apoptosis under the effect of tumor necrosis factor (TNF) released by infiltrating C-C chemokine receptor-2 (CCR2)^+^ inflammatory monocytes/macrophages, which also clear apoptotic FAPs.^26,27^ Both FAPs and macrophages are essential to orchestrate and complete appropriate myogenic repair from SCs, and the critical function of FAPs is thought to involve the secretion of appropriate growth factors and extracellular matrix, which enable SC proliferation, myogenic differentiation and myofiber assembly.^25,28,29^ As both muscle SCs and FAPs have osteogenic potential in vitro,^19,21^ the question of the cells of origin of NHOs has not been resolved.

Lineage-tracing experiments using the Cre-loxP system have been undertaken to identify which progenitors contribute to the inheritable genetic form of heterotopic ossification called fibrodysplasia ossificans progressiva (FOP). Unlike NHOs, which are frequent after severe CNS injury, FOP is an extremely rare disorder caused by a gain-of-function missense single nucleotide mutation in the bone morphogenetic protein (BMP) type I receptor gene *ACVR1*,^30^ resulting in a switch in ligand preference with aberrant activation of BMP signaling by activin-A instead of BMPs.^31^ Most lineage tracing experiments to identify the “cells of origin” of heterotopic ossifications were performed in mouse models of FOP expressing either a Cre recombinase-inducible *ACVR1*^R206H^ mutant that causes FOP in humans or a BMP transgene or with the insertion of a BMP-containing implant. In these mouse models of FOP, a variety of gene promoters have been used to drive Cre recombinase expression. Based on Cre recombinase driven by the *Pax7* (Paired Box 7) or *Cdh5* (Cadherin 5) gene promoters, which are specific to SCs and endothelial cells, respectively, it was concluded that neither SCs nor endothelial cells were the cells of origin of heterotopic ossifications in FOP.^32^ However, most of the gene promoters used in similar studies (e.g., Glast/*Slc1a3*, *Scx*, *Mx1*, *Tie2*, CD133/*Prom1* gene promoters)^32–34^ did not result in exclusive expression in mesenchymal progenitor cells, and the conclusion that FOP heterotopic ossifications were derived from mesenchymal cells was inferred by elimination of other possible cell candidates. In a mouse model of FOP with overexpression of a BMP4 transgene, it was recently shown that *Gli1*-expressing cells were integrated in HOs.^34^ However, BMP4 is a very strong osteogenic protein, and the relationship of the level of artificially high BMP4 expression by means of a transgene to SCI-induced NHOs remains to be demonstrated.^17,19^ Recently, a Cre mouse model with the *Pdgfra* gene promoter was used to demonstrate that HOs induced by BMP-2-supplemented Matrigel^35^ or by overexpressing the FOP-causing *ACVR1*^R206H^ mutant^36^ originated from mesenchymal cells expressing platelet-derived growth factor receptor-α (PDGFRα). However, while relevant models of FOP, artificial expression of osteogenic *ACVR1*^R206H^ or *ACVR1*^Q207D^ mutants or artificial overexpression of a BMP transgene or implantation of BMP-containing scaffolds utilized in these studies above are of little biological and clinical relevance to acquired NHOs developing after severe CNS injuries because NHOs occur with high prevalence in genetically normal patients of a broad range of ethnicities. In a mouse model of traumatic HOs induced by body burn and tenotomy and a model of FOP generated by induced Cre recombinase activation of the *ACVR1*^Q207D^ activating mutation, it was found that both burn-induced HOs in the tendon and subcutaneous FOP-induced HOs were derived from *Prrx1* gene-expressing mesenchymal progenitors.^37^ However, these HO models do not involve severe CNS trauma, which defines and triggers NHOs.^17,19^ Furthermore, while FOP flare-ups develop in many different types of tissues associated with muscles (e.g., skeletal muscles, tendons, ligaments, fascia, and aponeuroses),^38^ possibly due to the dominant effect of activating mutations of *ACVR1*, NHOs are mostly intramuscular in otherwise genetically normal patients.^1^ Therefore, which progenitor cells within skeletal muscles are the “cells of origin” for NHO development after SCI remains to be established. To do so, we crossed a Cre-inducible fluorescent Zoanthus green (ZsGreen) reporter mouse strain with either a strain with *Pax7-CreET2*-enabled specific expression in SCs^39^ or the *Prrx1-Cre* strain, which specifically expresses the Cre recombinase transgene in mesenchymal stem/progenitor cells under the control of the *Prrx1* gene enhancer.^40^ Finally, we sorted CD56^+^ myogenic progenitors and PDGFRα-expressing mesenchymal progenitors from human skeletal muscle surrounding NHOs after surgical excision and studied their osteogenic differentiation in vitro and in vivo in an ectopic bone model in immunodeficient mice.

## Results

### ZsGreen^+^ cells identify SCs and FAPs in the muscles of *Pax7*-CreERT2 and *Prrx1*-Cre mice

**Fig. 1:**
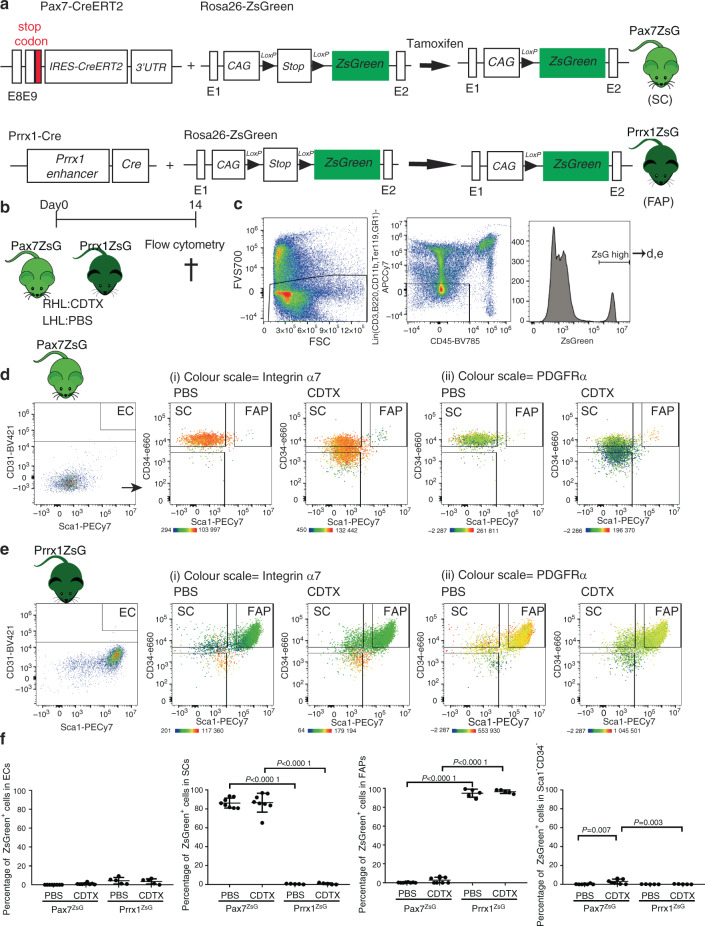
ZsGreen labels SCs and FAPs in skeletal muscles of the Pax7^ZsG^ and Prrx1^ZsG^ mice, respectively. **a** Schematic representation of tamoxifen-inducible Cre-dependent ZsGreen reporter induction. **b** *Pax7*^ZsG^ (*n* = 4/group) and *Prrx*1^ZsG^ (*n* = 3/group) mice were injected with CDTX in the hamstring muscle of the right hind limb (RHL) and PBS in the hamstring muscle of the left hind limb (LHL). Muscle cells were isolated 14 days after injection and subsequently stained for surface cell markers. **c** After gating on forward/side scatters and FVS700 dead cell exclusion, ZsGreen-high cells were gated from Lin^−^ CD45^−^ nonhematopoietic population in both (**d**) *Pax7*^ZsG^ and (**e**) *Prrx1*^ZsG^ mice and further subgrouped into CD31^+^Sca1^+^(EC), CD31^−^Sca1^−^CD34^+^(SCs), CD31^−^Sca1^+^CD34^+^ (FAPs), and Sca1^−^CD34^−^ populations. The color indicates the expression level of (**i**) integrin α7 and (**ii**) PDGFRα. Yellow-orange: high expression. Green: low expression. **f** Frequencies of ZsGreen^+^ cells in Sca1^+^CD31^+^ ECs, CD31^−^ Sca1^−^ CD34^+^ ITGA7^+^ PDGFRα^−^ SCs and CD31^−^ Sca1^+^ CD34^+^ ITGA7^−^ PDGFRα^+^ FAP populations from muscles of the *Pax7*^ZsG^ and *Prrx1*^ZsG^ mice following the gating strategy in Fig. S1. Each dot represents a separate mouse, and bars are the mean ± SD. The results of 8 mice per group were from 3 independent experiments performed over a period of 2 years with different flow cytometers. Significance was analyzed by one-way ANOVA with Tukey’s multiple comparison test

To determine whether NHOs are derived from muscle SCs and FAPs, we first assessed the specificity and efficiency of ZsGreen reporter expression in the *Pax7*-CreERT2; *Rosa26*-LoxP-STOP-LoxP-ZsGreen mice (abbreviated as *Pax7*^ZsG^ thereafter) and the *Prrx1*-Cre; *Rosa26*-LoxP-STOP-LoxP-ZsGreen mice (abbreviated as *Prrx1*^ZsG^) (Fig. 1a). CreERT2 was activated by daily gavage with tamoxifen for 4 days, after which mice were left to rest for an additional 2 weeks. The right hind limb hamstring muscle was then injected with cardiotoxin (CDTX) to cause muscle injury and initiate muscle regeneration. Fourteen days post-injury, muscles were harvested and dissociated for analyses of single muscle cell suspensions by flow cytometry (Fig. 1b). Within the CD45^−^ and hematopoietic lineage (Lin: Ter119, CD3ε, CD45R, CD11b, Gr1)-negative nonhematopoietic cell gate, endothelial cells were identified as CD31^+^ and stem cell antigen-1 (Sca1)-positive, SCs were identified as CD31^−^ CD34^+^ Sca1^−^ integrin α7 (ITGA7)^+^ PDGFRα^−^ and FAPs were identified as CD31^−^ CD34^+^ Sca1^+^ ITGA7^−^ PDGFRα^+^ (Fig. S1), in accordance with previous literature.^23,25,41^ The frequencies of SCs (CD31^−^ CD34^+^ Sca1^−^ ITGA7^+^ PDGFRα^−^), FAPs (CD31^−^ CD34^+^ Sca1^+^ ITGA7^−^ PDGFRα^+^) and endothelial cells (CD31^+^ Sca1^+^) were similar between the CDTX-injured and noninjured muscles in both the *Pax7*^ZsG^ and *Prrx1*^ZsG^ strains 14 days post-injury (Table S1).

We subsequently gated ZsGreen^+^ cells within the hamstring muscles of these mice (Fig. 1c). In the vehicle-injected muscles of the *Pax*7^ZsG^ mice, all ZsGreen^+^ cells had the typical SC phenotype (CD45^−^ Lin^−^ CD31^−^ CD34^+^ Sca1^−^ ITGA7^+^ PDGFRα^−^) with no ZsGreen^+^ cells in the endothelial gate and very few in the FAP gate (Fig. 1d - PBS). In the CDTX-injured muscle, the distribution of ZsGreen^+^ cells was similar but with the emergence of CD45^−^ Lin^−^ CD34^−^ Sca1^−^ ITGA7^+^ PDGFRα^−^ cells derived from ZsGreen^+^ SCs (Fig. 1d—CDTX). These cells are likely differentiating myoblasts.

Conversely, in the vehicle-injected contralateral muscle of the *Prrx1*^ZsG^ mice, most ZsGreen^+^ cells had the typical FAP phenotype with no ZsGreen^+^ cells in the endothelial gate and very few in the typical SC gate (Fig. 1e PBS). The phenotypes of ZsGreen^+^ cells were the same in the CDTX-injured muscle of the *Prrx1*^ZsG^ mice (Fig. 1e—CDTX).

In addition to the high specificity of these lineage tracing strategies, the recombination efficiency was also very high, with over 90% of SCs and FAPs labeled in the muscle of the *Pax7*^ZsG^ and *Prrx1*^ZsG^ strains, respectively (Fig. 1f). Importantly, Cre-mediated recombination in the *Pax7*^ZsG^ and *Prrx1*^ZsG^ mice did not affect subsequent NHO development compared to that in the C57BL/6 mice, as measured by microcomputerized tomography (µCT) or hematoxylin-eosin staining, which clearly showed the presence of necrotic and fibrotic muscle together with inflammatory infiltrate and sporadic bony nodules 28 days after injury in both strains, as we previously reported in C57BL/6 mice^13,20,21,42^ (Fig. S2). Altogether, these experiments confirmed that ZsGreen was specifically expressed in the targeted muscle cell populations in both the *Pax7*^ZsG^ and *Prrx1*^ZsG^ strains without altering NHO development, thus illustrating their suitability for lineage tracing of SCs and FAPs in vivo.

### NHOs are not derived from *Pax7*-expressing SCs

**Fig. 2:**
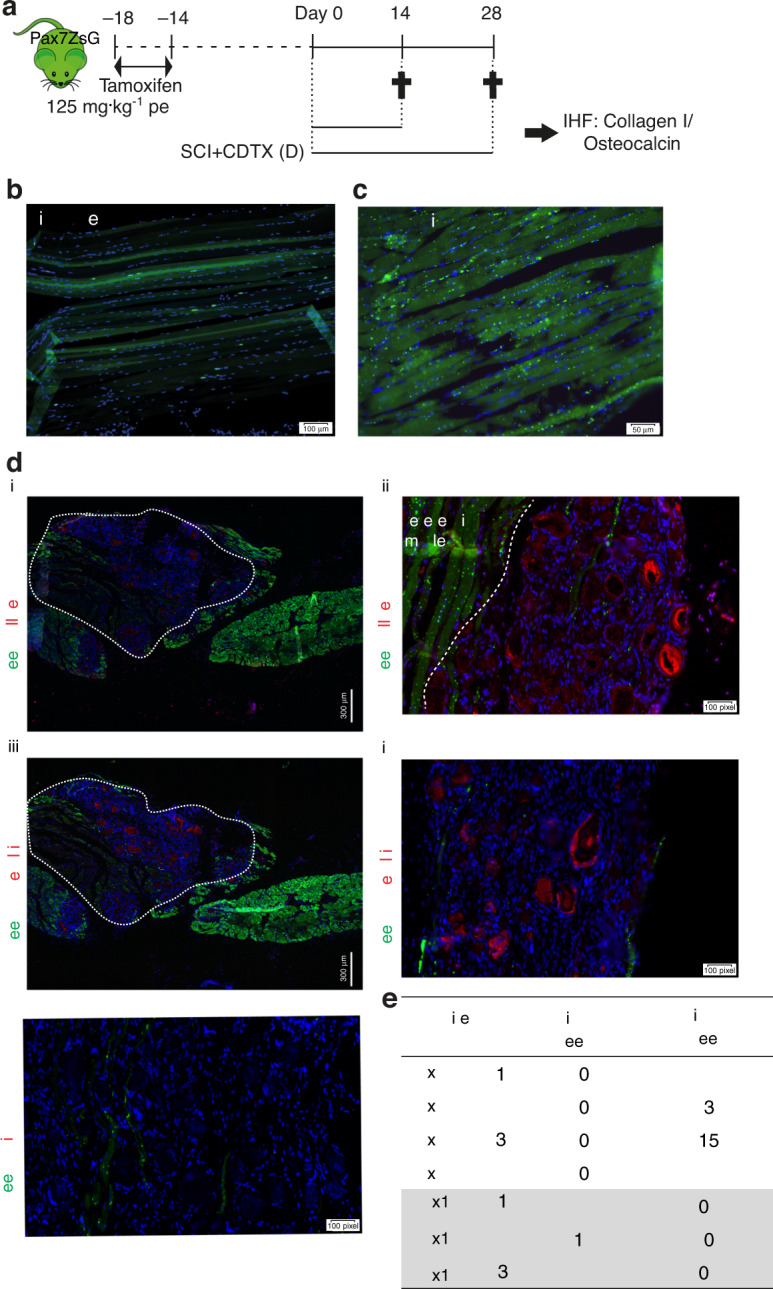
NHOs are not derived from *Pax7* expressing SCs. **a** ZsGreen expression in *Pax7*^ZsG^ mice was induced by tamoxifen treatment for 4 days. Two weeks after tamoxifen treatment, the mice received an intramuscular injection of CDTX with or without SCI. Muscle samples were harvested 14 or 28 days post-injury. Representative IHF images illustrating the distribution of ZsGreen^+^ SC-derived cells in (**b**) uninjured muscle and (**c**) regenerated injured muscle 14 days post-CDTX injection in the *Pax7*^ZsG^ mice without SCI (*n* = 3 mice/group). Scales bars: (**b**) 100 μm, (**c**) 50 μm. **d** Representative images from the *Pax7*^ZsG^ mice with SCI and CDTX-mediated muscle injury 28 days post-surgery. **i** IHF staining illustrating that ZsGreen^+^ cells are present among areas of regenerating muscle and largely absent from areas of NHO development. White dashed lines indicate the boundary between regenerating muscle and fibrotic area containing NHOs stained red (i–ii) for collagen I and (iii-iv) osteocalcin. Nuclei were stained by DAPI staining (blue). A negative control was performed using rabbit isotype IgG (iv). Scale bars: (**d**) (**i, iii**) 300 μm, (**ii, iv, v**) 50 μm. **e** Number of osteocalcin^+^ NHOs intercalated with ZsGreen^+^ cells or without ZsGreen^+^ cells in both *Pax7*^ZsG^ (*n* = 4 mice, total 44 osteocalcin^+^ NHOs counted) and *Prrx*1^ZsG^ mice (*n* = 3 mice, total 30 osteocalcin^+^ NHO counted). Statistical differences were determined using Fisher’s exact test (*P* < 10^−4^)

ZsGreen expression was then examined on frozen longitudinal sections of uninjured muscle, repaired muscle and NHOs from the *Pax7*^ZsG^ mice (Fig. 2a). In noninjured muscles from the *Pax7*^ZsG^ mice, ZsGreen was expressed in a discrete population of small cells distributed along myofibers typical of SCs (Fig. 2b). Some myofibers were also ZsGreen^+^, likely reflecting their natural turnover from ZsGreen^+^ SCs over a period of 4 weeks after tamoxifen induction. Fourteen days after CDTX-induced muscle injury, in the absence of SCI, all myofibers were ZsGreen^+^, as anticipated, illustrating muscle regeneration from ZsGreen^+^ SCs (Fig. 2c).

In the cohort of mice that underwent spinal cord transection (SCI) and CDTX-mediated muscle injury, frozen sections were stained with specific anti-collagen I [Fig. 2d(i-ii)] and anti-osteocalcin antibodies [Fig. 2d(iii-iv)]. An example of a nonimmune IgG negative control for anti-collagen I and anti-osteocalcin antibodies is shown in Fig. 2d(v). Immunohistofluorescence (IHF) confirmed ZsGreen^+^ cells within areas of regenerating muscle that contained neoformed ZsGreen^+^ myofibers. Most importantly, ZsGreen^+^ cells were largely absent among areas of fibrotic muscle and NHO nodules. ZsGreen^+^ cells were not intercalated among the collagen I^+^ bone matrix or osteocalcin^+^ osteoblasts on NHO nodules. Quantification of NHOs through four different *Pax7*^ZsG^ mice showed that none of the 44 osteocalcin^+^ NHOs contained ZsGreen^+^ cells (Fig. 2e). This finding demonstrates that NHOs following SCI are not derived from ZsGreen^+^ SCs.

### NHOs are derived from *Prrx1*-expressing FAPs

**Fig. 3:**
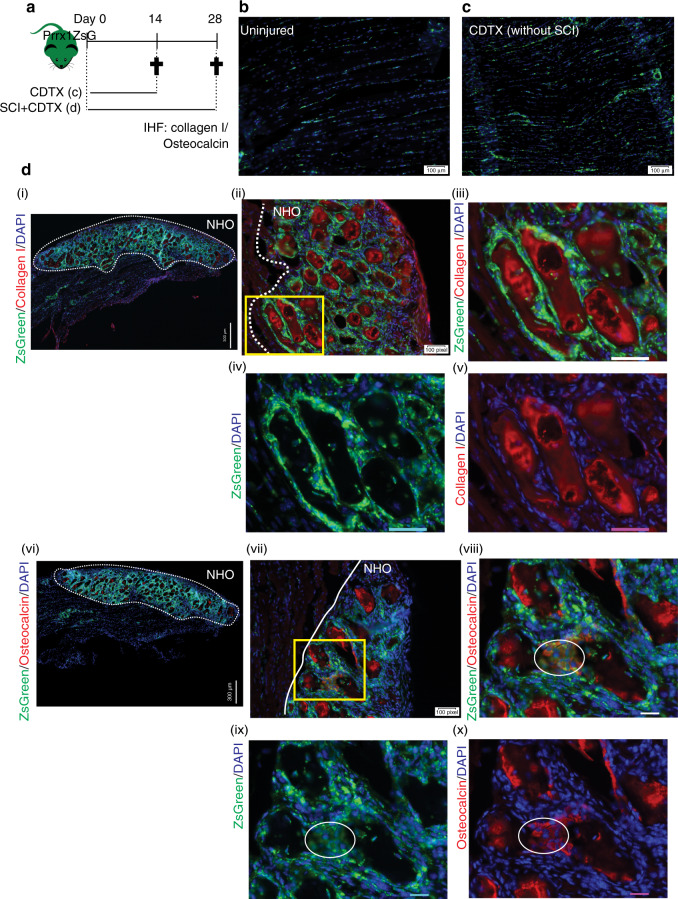
NHOs are derived from *Prrx1* expressing FAPs. **a** *Prrx*1^ZsG^ mice received an intramuscular injection of CDTX with or without SCI, and muscle samples were harvested at the indicated time points and processed for IHF. Representative images illustrating the distribution of ZsGreen^+^ FAP-derived cells in (**b**) uninjured muscle and **c** regenerated injured muscle 14 days post-CDTX injection in the *Prrx1*^ZsG^ mice without SCI (*n* = 3 mice/group). **d** Representative images from the *Prrx1*^ZsG^ mice with SCI and CDTX-mediated muscle injury 28 days post-surgery: IHF illustrating the colocalization of ZsGreen^+^ FAP-derived cells with (**i**–**v**) collagen I^+^ matrix (red) or (**vi-x**) osteocalcin^+^ osteoblasts (red). (**iii-v**) are enlarged images of the yellow box in (**ii**), whereas (**viii-x**) are enlarged images of the yellow box in (**vii**). The white dashed line indicates the boundary between regenerating muscle and the fibrotic area containing NHOs. Nuclei stained in DAPI (blue). White circles indicate osteocalcin^+^ osteoblasts that also express ZsGreen. Scale bars: (**b**, **c**) 100 μm; (**d**) (i) 300 μm (**ii, vii**) 100 μm (**iii-v, viii-x**) 50 μm

ZsGreen expression was also examined on frozen longitudinal sections of uninjured muscle, repaired muscle and NHOs from the *Prrx1*^ZsG^ mice (Fig. 3a). In the uninjured muscle, reticulated ZsGreen^+^ cells were scattered in the interstitium along myofibers (Fig. 3b), a typical distribution and morphology of FAPs.^25^ Importantly, in the regenerating CDTX-injured muscle (without SCI), ZsGreen^+^ cells were distributed similarly, and most importantly, they did not contribute to neoformation of myofibers (Fig. 3c), in concordance with the literature.^25^

The *Prrx1*^ZsG^ mice that underwent SCI and CDTX-mediated muscle injury also developed NHOs (similar to *Pax7*^ZsG^ mice that underwent the same treatment) (Fig. S2). However, in sharp contrast to those of the *Pax7*^ZsG^ mice, all fibrotic areas in the SCI + CDTX-injured muscles of the *Prrx1*^ZsG^ mice were intensely labeled by ZsGreen, particularly around collagen I^+^ and osteocalcin^+^ NHO nodules [Fig. 3d(ii-x)]. We counted 30 osteocalcin^+^ NHOs from three *Prrx1*^ZsG^ mice, and all of them were intercalated with ZsGreen^+^ cells (Fig. 2e). At higher magnification, differentiating osteocalcin^+^ osteoblasts (red) were also ZsGreen^+^ [Fig. 3d(vii-x) circles], suggesting that NHOs are derived from *Prrx1*-expressing mesenchymal progenitor cells rather than SCs. To confirm that NHOs are formed from *Prrx1*-expressing FAPs and not by *Pax7*-expressing SCs, we performed Fisher’s exact test on all NHO nodules manually counted on tissue sections from 4 *Pax7*^ZsG^ mice and 3 *Prrx1*^ZsG^ mice (Fig. 2e). The absence/presence of ZsGreen in NHO nodules from these 2 strains was significantly different (*P* < 10^−4^). An additional Fisher’s exact test detected ZsGreen fluorescence in NHOs of 3 mice of the 3 *Prrx1*^ZsG^ mice containing NHOs, whereas ZsGreen was absent from all NHOs in the 4 *Pax7*^ZsG^ mice, which demonstrated that ZsGreen was differentially distributed in the NHOs from the two strains (*P* = 0.028 6). Together, these results show that NHOs are derived from *Prrx1*-expressing mesenchymal progenitor cells but not from SCs.

### Mesenchymal *Prrx1*^+^ cells do not circulate in blood after SCI

Parabiosis models have been previously used to track circulating cells that contribute to heterotopic ossifications (HOs). A ubiquitous green fluorescent protein (GFP) reporter mouse was joined with a wild-type mouse, with the wild-type mouse receiving Achilles tendon tenectomy and dorsal burn injury to induce HO formation. Circulating GFP^+^ cells from the GFP-expressing parabiont contributed to HO development in the wild-type parabiont 28 days after tenectomy and burn injury.^43^ However, a more recent parabiosis study suggests otherwise: HO induced by inserting BMP2-supplemented Matrigel matrix did not contain cells derived from PDGFRα^+^ FOPs recruited from the other parabiont via the shared circulation, suggesting that the cells of origin of BMP2-induced HOs are not recruited via the circulation.^35^

To clarify the dichotomy of these two previous publications, we first investigated whether SCI activates the expression of osteogenic BMPs and BMP signaling in injured muscles. SCI did not increase the expression of *Bmp2*, *Bmp4* or *Bmp7* RNA in paraplegic hindlimb muscles (Fig. S3a). In contrast, either SCI or muscle injuries decreased the expression of osteogenic BMPs. We also investigated the effect of daily treatment with LDN-193189, a potent inhibitor of BMP type I receptor serine kinases that has been shown to be effective at inhibiting HO development in the mouse FOP model driven by the *ACVR1*^Q207D^ missense mutation^44^ and in the burn-induced rat model of ossifying tendinopathy.^45^ While LDN-193189 significantly inhibited mineralization of mouse bone marrow-derived mesenchymal stromal cells (BM-MSCs) cultured in osteogenic conditions (Fig. S3b), treatment of mice with LDN-193189 either in the first two weeks of the injury or during the maturation phase of NHOs between weeks 2 and 3 post-injury had no effect on NHO development (Fig. S3c-d). As BMPs are known to induce endochondral ossification, we stained sections of CDTX-injured muscles at days 7, 14 and 21 following SCI with safranin O to span the time course of NHO development in mice (Fig. S4). Although occasional mast cells brightly stained by safranin O could be detected in the developing NHO, there was no evidence of cartilage matrix in any NHO examined at any time point (*n* = mice per time point). Therefore, unlike the *ACVR1*^Q207D^ FOP model and the burn-induced ossifying tendinopathy model, the BMP signaling inhibitor LDN-193189 was unable to reduce NHO development after SCI, suggesting that NHO development following SCI is not as BMP-dependent as the two other processes of HO development and does not involve endochondral ossification.

**Fig. 4:**
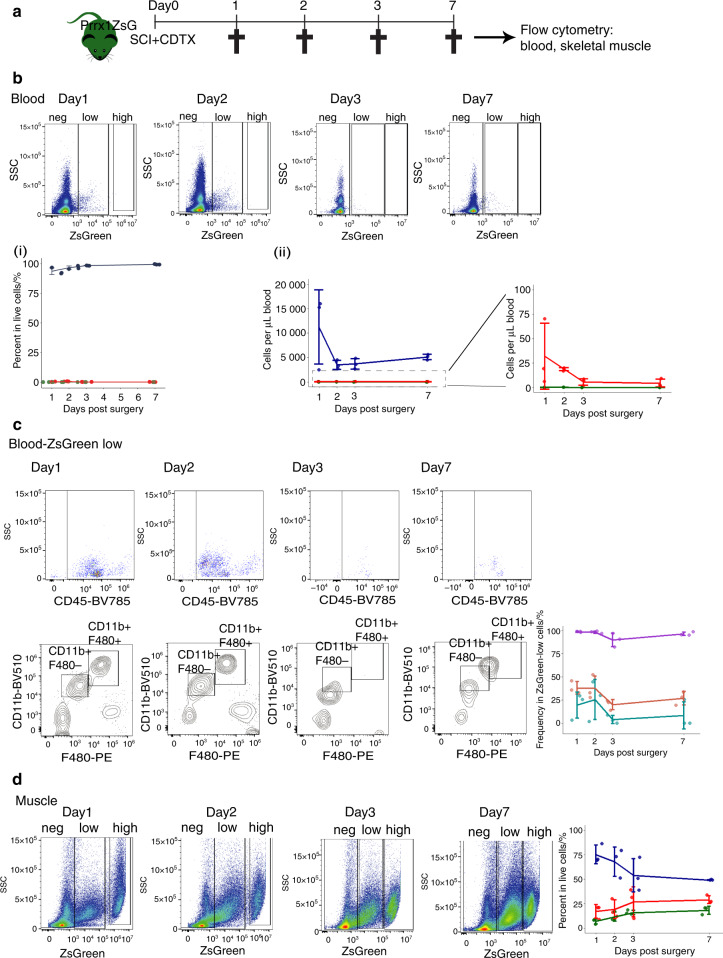
Absence of ZsGreen^high^ mesenchymal cells in the circulation of the *Prrx1*^ZsG^ mice after SCI and muscle injury. **a** *Prrx*1^ZsG^ mice received SCI and an intramuscular injection of CDTX. Peripheral blood and skeletal muscles were collected at 1, 2, 3, and 7 days post-surgery and analyzed by flow cytometry (*n* = 3 mice/group). **b** FVS700^−^ live cells from peripheral blood were gated based on the intensity of ZsGreen fluorescence into ZsGreen negative (blue lines), low (red lines) and high groups (green lines). The frequency of these three populations is represented as (**i**) the frequency of live cells in blood and (**ii**) the number of cells per μl blood. **c** The ZsGreen-low cells were further gated for expression of CD45, CD11b and F480. The frequency of CD45^+^ leukocytes (purple line), CD45^+^ CD11b^+^ F4/80^+^ monocytes (red line) and CD45^+^ CD11b^+^ F4/80^−^ granulocytes (blue line) among circulating ZsG^low^ cells was plotted. **d** Live cells from injured muscle from the same *Prrx1*^ZsG^ mice were used as a reference for ZsGreen fluorescence intensity, confirming the presence of numerous ZsGreen^high^ cells in muscle. ZsGreen negative (blue lines), low (red lines) and high (green lines). Each dot represents a separate mouse. Bars represent the mean ± SD. There was no significant difference between the time points as determined by one-way ANOVA with Tukey’s multiple comparison test

We next investigated whether *Prrx1*^+^ mesenchymal cells at the origin of NHOs could be derived from the circulation. As parabiosis experiments are forbidden for ethical reasons in Australia, we investigated whether ZsGreen^+^ cells were detectable in the circulation of *Prrx1*^ZsG^ mice 1, 2, 3 and 7 days following SCI and CDTX-mediated muscle injury (Fig. 4b, c). Flow cytometry of blood nucleated cells showed that there was a ZsGreen^low^ population that mostly included CD45^+^ CD11b^+^ F4/80^+^ monocytes and CD45^+^ CD11b^+^ F4/80^−^ populations (Fig. 4c), but the intensity of ZsGreen fluorescence in these phagocytes was two full log_10_ units lower than the ZsGreen fluorescence intensity of FAPs in the muscle (Fig. 4d). Therefore, it is likely that the low ZsGreen fluorescence of blood monocytes and granulocytes is due to phagocytosis of ZsGreen (possibly packaged in extracellular vesicles^46,47^) produced by *Prrx1*-expressing mesenchymal progenitor cells and their progenies. Importantly, despite analyzing over 10^6^ blood leukocytes per mouse, we could not detect any circulating CD45^−^ Lin^−^ CD31^−^ Sca1^+^ mesenchymal progenitor cells or cells with ZsGreen fluorescence intensity as high as FAPs in the muscle of the *Prrx1*^ZsG^ mice (Fig. 4b, d). As another potential source of “mobilized” mesenchymal cells is the bone marrow, we transplanted 90 000 stromal cells enriched from the bone marrow of the *Prrx1*^ZsG^ mice by magnetic activated cell depletion of CD45^+^ leukocytes and Ter119^+^ erythroid cells using the EasySep mouse stromal cell enrichment kit (Stem Cell Technologies). Following this enrichment step, 11.7% of these bone marrow stromal cells were ZsG^bright^ with the classic CD45^−^ Lin^−^ CD31^−^ Sca1^+^ mesenchymal progenitor cell phenotype. These cells were transplanted i.v. into three C57BL/6 recipients 24 h post-SCI and CDTX muscle injury of the recipients. Muscles were analyzed by histofluorescence 21 days later, and none of the CDTX-injured muscles contained any ZsGreen-positive cells (result not shown). Therefore, while we cannot completely exclude the possibility that some of the *Prrx1*-expressing ZsGreen^+^ cells associated with NHOs following SCI may come from the circulation, these would be extremely rare compared to the abundance of *Prrx1*-expressing ZsGreen^+^ FAPs already present in the muscle prior to injury. Therefore, NHOs are most likely derived from muscle-resident FAPs.

### Spinal cord injury causes upregulated PDGFRα expression on FAPs regardless of muscle injury

To better understand the effect of SCI on muscle FAPs, we followed PDGFRα expression on muscle cells. Surprisingly, PDGFRα expression was significantly upregulated on CD45^−^ Lin^−^ CD31^−^ Sca1^+^ CD34^+^ FAPs at 7 and 14 days after SCI in both the CDTX-injured and contralateral noninjured muscles (Fig. S5a–c). However, no expression of PDGFRα was noted on CD45^−^ Lin^−^ CD31^−^ Sca1^−^ CD34^+^ SCs in any of the experimental conditions tested (Fig. S5d). Although it remains to be determined whether this upregulation of PDGFRα expression has a functional role in driving NHO development, this finding suggests that SCI can alter or reprogram FAP function, as shown by PDGFRα expression.

### Spinal cord injury leads to reduced apoptosis and persistent proliferation of FAPs following muscle injury

**Fig. 5:**
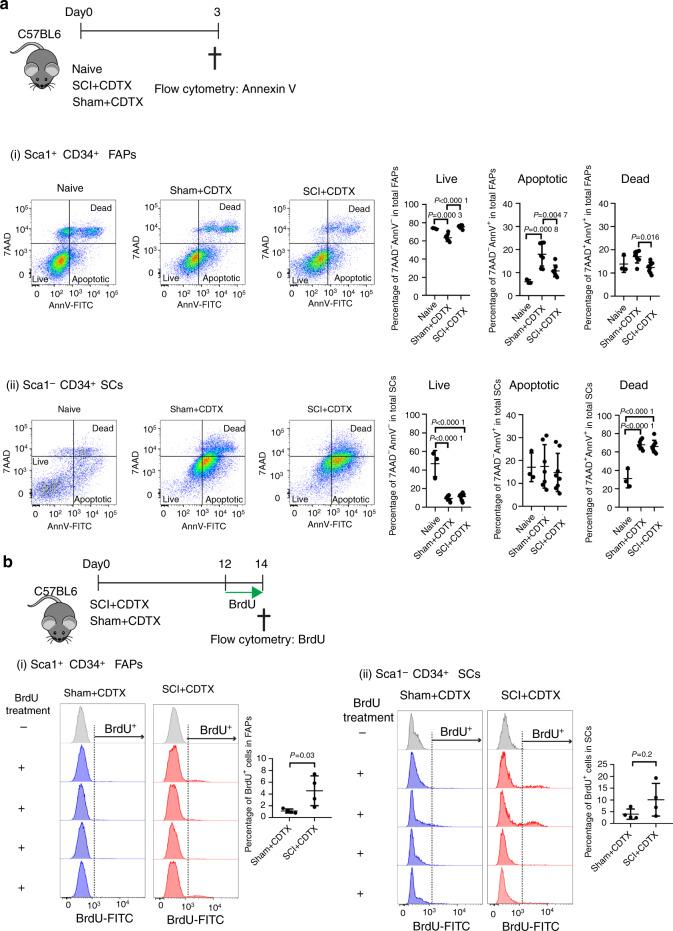
SCI leads to decreased apoptosis and persistent proliferation of FAPs in injured muscles. **a** Naïve C57BL/6 mice underwent SCI or sham surgery plus intramuscular injection of CDTX. Muscle cells were isolated 3 days later. Apoptotic cells were subsequently analyzed by Annexin V (AnnV) and 7-amino-actinomycin D (7AAD) staining by flow cytometry. (i) CD45 Lin^−^ CD31^−^Sca1^+^ CD34^+^ ITGA7^−^ FAPs and (ii) CD45 Lin^−^ CD31^−^Sca1^−^ CD34^+^ ITGA7^+^ SCs were gated. AnnV and 7AAD staining further distinguished cells as live (7AAD^−^AnnV^−^), apoptotic (7AAD^−^AnnV^+^) and postapoptotic dead (7AAD^+^AnnV^+^) in both FAP and SC populations. The percentages of live, apoptotic and dead cells in the total FAP or SC populations are presented as the mean ± SD (*n* = 3, 7, and 8 in naïve, sham+CDTX, and SCI + CDTX, respectively). Each dot represents a separate mouse. Significance was calculated by one-way ANOVA with Tukey’s multiple comparison test. **b** C57BL/6 mice underwent SCI or sham surgery plus intramuscular injection of CDTX. Mice were given drinking water containing BrdU together with BrdU i.p. injection (twice daily) from day 12 to 14. One SCI + CDTX and 1 sham+CDTX mouse were not treated with BrdU and used as a negative control for anti-BrdU staining. On day 14, muscle cells were isolated, and BrdU staining was analyzed in CD45 Lin^−^ CD31^−^Sca1^+^ CD34^+^ ITGA7^−^ FAPs and CD45 Lin^−^ CD31^−^Sca1^−^ CD34^+^ ITGA7^+^ SCs by flow cytometry. The percentage of BrdU^+^ cells in the total FAP or SC population is presented as the mean ± SD. Each dot represents a separate mouse. Significance was calculated by a two-sided Mann–Whitney test

Sequential induction of FAP proliferation followed by apoptosis is crucial for effective muscle repair, as either increased proliferation or decreased apoptosis can result in overexpansion of FAPs in the regenerating muscle, resulting in fibrosis.^27^ To examine whether the dynamics of FAP proliferation and apoptosis are perturbed during NHO formation, we first compared by flow cytometry the frequencies of apoptotic cells within FAP and SC populations three days post-injuries, a time point at which FAP apoptosis peaks in the regenerating muscle^27^ (Fig. 5a). We stained muscle cell suspensions with annexin V (AnnV) to detect the early stage of apoptosis together with nonmembrane permeable DNA dye 7-amino-actinomycin D (7AAD) to identify live cells (AnnV^−^ 7AAD^−^), apoptotic cells (AnnV^+^ 7AAD^−^) and postapoptotic dead cells (AnnV^+^ 7AAD^+^). Within the Lin^−^ CD45^−^ CD31^−^ Sca1^+^ CD34^+^ ITGA7^−^ FAP population, muscle injury alone (sham + CDTX) led to a 17% reduction in live FAPs and a 3.2-fold increase in apoptotic FAP frequency compared to that of naïve muscle, as previously reported^27^ [Fig. 5a(i)]. However, in mice that had undergone SCI together with muscle injury, the frequencies of live and apoptotic FAPs were reversed and similar to those found in naïve mice. With respect to the Lin^−^ CD45^−^ CD31^−^ Sca1^−^ CD34^+^ ITGA7^+^ SC population, CDTX injury led to an 81% reduction in live SCs and a 2.6-2.7-fold increase in dead SCs regardless of the presence or absence of SCI [Fig. 5a(ii)].

We next measured muscle progenitor proliferation by in vivo 5-bromo-2’-deoxyuridine (BrdU) incorporation 14 days post surgeries. To ensure sufficient incorporation of BrdU into newly synthesized DNA during cell proliferation, we treated the mice with BrdU two days prior to tissue harvest. The percentage of proliferative BrdU^+^ FAPs was 4.08-fold higher in the SCI + CDTX group than in the sham+CDTX group [Fig. 5b(i)], while there was no significant difference in the proliferation of SCs [Fig. 5b(ii)]. These results confirm that SCI deregulates the coordinated proliferation and apoptosis of FAPs in injured muscles.

### Human PDGFRα^+^ cells from muscles surrounding NHOs support both in vitro and in vivo bone formation

**Fig. 6:**
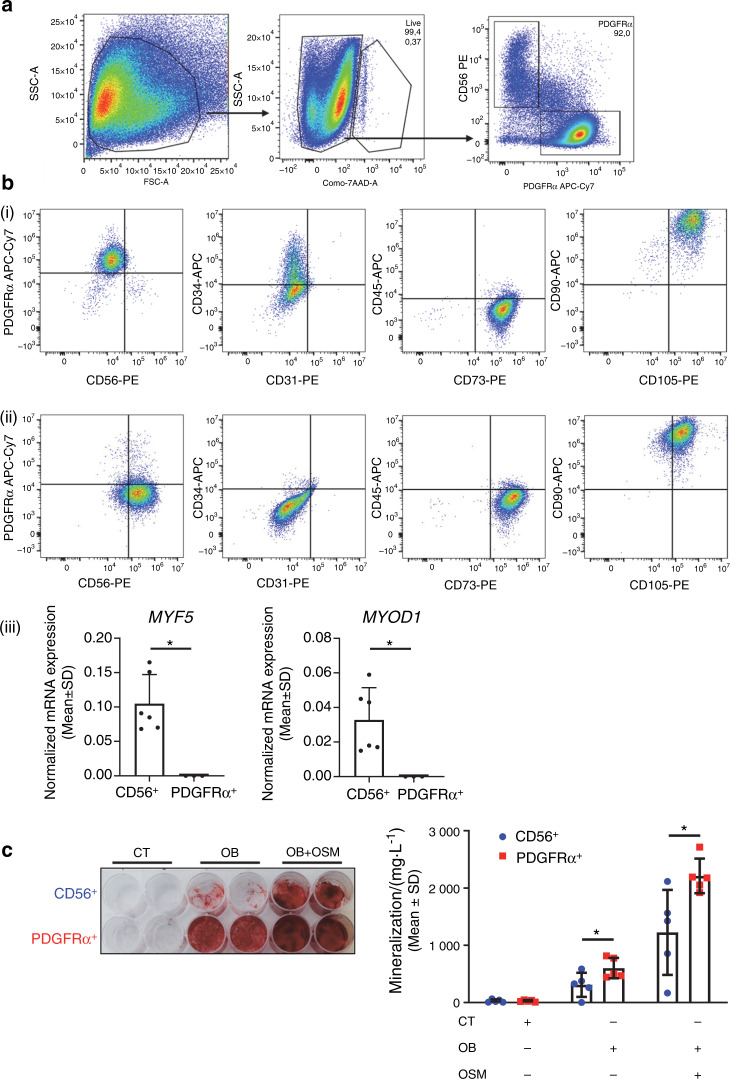
Human PDGFRα^+^ cells isolated from muscles surrounding NHOs support in vitro bone formation. **a** Flow cytometry gating strategy of PDGFRα^+^ and CD56^+^ cell subpopulations isolated from muscle surrounding NHOs. **b** Representative surface marker characterization by flow cytometry: CD56, PDGFRα, CD31, CD34, CD45, CD73, CD90, and CD105, (**i**) CD56^+^ population, (**ii**) PDGFRα^+^ population, (**iii**) Normalized mRNA expression of *MYF5* and *MYOD1* by qRT-PCR expressed as the mean ± SD (CD56^+^ *n* = 6; PDGFRα^+^ *n* = 3). **c** In vitro osteoblastic differentiation assay seeded with CD56^+^ or PDGFRα^+^ cells isolated from muscles surrounding human NHOs. **(i**) All cells were cultured in control medium (CT) or osteogenic medium alone (OB) or were supplemented with human OSM (100 ng·mL^−1^) (OB + OSM) for 14 days followed by Alizarin Red S staining. **c**(**ii**) Quantification of calcium mineralization expressed as the mean ± SD (*n* = 5). *P < 0.05, two-sided nonparametric Mann–Whitney U test

To validate the involvement of muscle-resident mesenchymal cells in human NHO pathology, we collected surgical residues of NHOs after resection surgery in twelve patients with SCI, TBI and stroke. Cells were isolated from the muscle tissue surrounding the resected NHOs using mechanical dissociation and enzymatic digestion and expanded in culture. PDGFRα^+^ and CD56^+^ cells were then isolated by fluorescence-activated cell sorting (Fig. 6a). The PDGFRα^+^ population displayed a classical mesenchymal phenotype: CD31^−^ CD45^−^ CD73^+^ CD90^+^ CD105^+^ [Fig. 6b(i)]. Interestingly, PDGFRα^+^ cells showed heterogeneous expression of CD34, a common marker of hematopoietic and angiogenic progenitor cells [from 2% to 46.1%; Fig. 6b(ii)]. As previously described,^48^ CD56^+^ cells were CD73^+^ CD90^+^ CD105^+^ [Fig. 6b(ii)] and expressed the myogenic regulatory transcription factors *MYF5* and *MYOD1* [Fig. 6b(iii)], whereas sorted PDGFRα^+^ cells did not.

In vitro osteogenic differentiation assays showed that PDGFRα^+^ cells exhibited a higher osteoblastic differentiation capacity than CD56^+^ cells (Fig. 6c), and osteogenic differentiation was enhanced by the addition of 100 ng·mL^−1^ recombinant human OSM (Fig. 6c), a key inflammatory mediator of NHO formation, as we previously reported.^21^

**Fig. 7:**
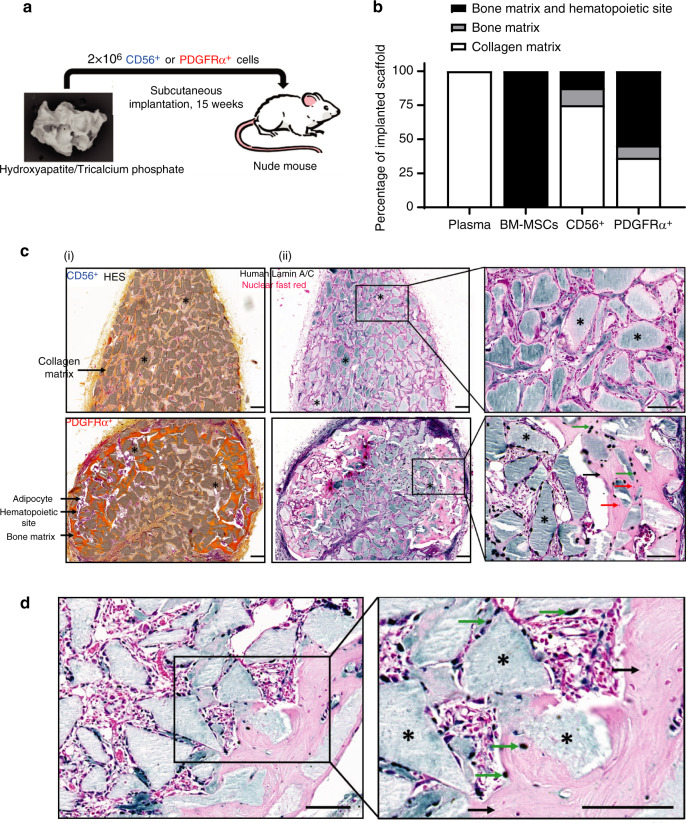
Human PDGFRα^+^ cells isolated from muscles surrounding NHO support in vivo bone formation. **a** Schematic representation of the in vivo osteogenic assay. Hydroxyapatite/calcium phosphate plasma scaffolds were seeded either with 2 × 10^6^ CD56^+^ cells, 2 × 10^6^ PDGFRα^+^ cells sorted from muscles surrounding NHOs, 2 × 10^6^ human BM-MSCs or without cells (“plasma” negative control) and subcutaneously implanted in the backs of nude mice for 15 weeks. **b** Percentage of scaffolds containing either collagen matrix alone, bone matrix alone, or bone matrix associated with hematopoietic colonization. Human plasma (*n* = 4 donors), BM-MSCs (*n* = 6 donors), CD56^+^ cells (*n* = 8; 4 donors, 2 implants/donor) and PDGFRα^+^ cells (*n* = 11; 6 donors, 2 implants/donor for 5 donors and 1 implant for one donor). **c(i**) Representative images of hematoxylin-eosin-safranin (HES) staining of CD56^+^ and PDGFRα^+^ cell-seeded implant sections. Nuclei are stained purple, cell cytoplasm is stained pink, and collagen fibers are stained orange. *ː hydroxyapatite. Magnification 10X; scale bar = 100 µm. **c**(**ii**) Specific human Lamin A/C staining of representative CD56^+^ and PDGFRα^+^ cell-seeded implant sections. *: hydroxyapatite scaffold; black arrows: bone matrix; green arrow: human osteocytes; red arrows: mouse osteocytes. Magnification ×10 and ×20; scale bar = 100 µm. **d** Osterix/SP7 staining of a representative PDGFRα^+^ cell-seeded implant section. *: hydroxyapatite scaffold; black arrows: bone matrix; green arrows: osterix^+^ osteoblasts. Magnification ×20 and ×40; scale bar = 100 µm

We then investigated whether cells sorted from muscles surrounding human NHO were able to support in vivo heterotopic bone formation in immunodeficient mice. PDGFRα^+^ and CD56^+^ cells were independently seeded into plasma-clotted hydroxyapatite/calcium phosphate scaffolds and implanted subcutaneously into the backs of nude mice (Fig. 7a). Unseeded plasma-clotted scaffolds were used as a negative control, and BM-derived mesenchymal stromal cell (BM-MSC)-seeded plasma-clotted scaffolds were used as positive controls. Fifteen weeks after implantation, scaffolds were collected for histological analysis. As expected, no bone tissue was observed in the control plasma group [Fig. S6a(i)], while all BM-MSC-seeded implants exhibited bone matrix and hematopoietic foci, as detected by hematoxylin, eosin and safranin staining (HES) [Fig. S6a(ii)]. Six of 11 implants (54.5%) with NHO muscle PDGFRα^+^ cells showed mature bone matrix containing osteocytes together with a hematopoietic marrow, demonstrating the formation of ectopic bone with functional hematopoietic BM [Fig. 7b, c(i)], as evidenced by the presence of numerous mature megakaryocytes (Fig. S6b). In contrast, only 12.5% of scaffolds seeded with CD56^+^ cells contained bone matrix deposition alone, and 12.5% showed both bone matrix development and hematopoietic colonization [Fig. 7b, c(i)], suggesting a much higher osteogenic potential of PDGFRα^+^ mesenchymal cells derived from human muscle surrounding NHOs. To analyze the origin of bone-forming cells in these implants, we investigated the expression of human-specific lamin A/C by immunohistochemistry. Figure 7c(ii) highlights that very few human cells were observed within CD56^+^ cell seeded scaffolds, whereas PDGFRα^+^ cell seeded implants exhibited numerous human lamin A/C^+^ osteocytes within the bone matrix [Fig. 7c(ii)], demonstrating that human PDGFRα^+^ cells actively participate in the formation of heterotopic bone. Of note, no human cells were detected within hematopoietic foci, confirming that hematopoietic cells that colonized these heterotopic bone formations were of murine origin (Fig. S6b). Finally, PDGFRα^+^ cell implants exhibited numerous osterix^+^ osteoblasts; most of them were located near hydroxyapatite particles and within the bone matrix (Fig. 7d). Thus, mesenchymal PDGFRα^+^ cells isolated from muscles surrounding human NHOs have a substantially higher capacity to support mature hemogenic bone formation than myogenic CD56^+^ cells. These data are concordant with the hypothesis that muscle-resident PDGFRα^+^ cells are key actors in the onset of NHOs in both humans and mice.

## Discussion

Although NHOs develop mostly in peri-articular muscles in humans,^1,2^ their cellular origin remains unclear. Using *Pax7*^ZsG^ mice that specifically trace muscle SCs and their myogenic progenies, we clearly demonstrate that NHOs developing after SCI in injured muscles in mice are not derived from muscle SCs. We also reveal that NHOs only develop in areas of the injured muscle where SCs fail to regenerate myofibers, with NHOs only found in areas where ZsGreen-labeled SC-derived myocytes or myofibers are completely absent. Likewise, CD56^+^ muscle progenitors sorted from the muscles surrounding NHOs from SCI patients do not effectively generate ectopic bones when transplanted into permissive immunodeficient mice. Therefore, although NHOs almost always develop in muscles, they are not the product of muscle SC transdifferentiation. In contrast, we found focal accumulation and osteoblastic differentiation of the *Prrx1*-expressing PDGFRα^+^ mesenchymal progenitor cells among areas of NHO development in the *Prrx1*^*ZsG*^ mice. This finding suggests that SCI perturbs normal or early proliferation followed by apoptosis of FAPs in the injured muscle with continued proliferation and absence of apoptosis of FAPs. High magnification of NHO sections in the *Prrx1*^ZsG^ mice also revealed that osteocalcin^+^ osteoblasts and osteogenic progenitors contained in the developing NHOs were labeled by ZsGreen and thus derived from the *Prrx1*-expressing mesenchymal progenitor cells that shared the same antigenic profile as muscle FAPs. Likewise, over half of PDGFRα^+^ mesenchymal progenitors sorted from the muscles surrounding NHOs from SCI patients effectively formed ectopic bones when transplanted into permissive immunodeficient mice. One of 8 preparations of CD56^+^ muscle progenitor cells from these NHO biopsies could form ectopic bones with hematopoietic marrow when implanted in mice. Although it remains unclear whether this was caused by contaminating mesenchymal cells in the sorted population, our results suggest that in both mice and humans, NHOs are derived from mesenchymal progenitor cells and not from myogenic progenitor cells such as SCs.

The *Pax7*Cre^ERT2^ model we employed has been used by many groups to specifically target and trace SCs in muscles following injury,^39,49,50^ which validates our use of *Pax7*^ZsG^ in our study. While there is no marker that is exclusively restricted to muscle FAPs, it is well known that *Prrx1*Cre (often called Prx1-Cre) targets and labels mesenchymal stromal cells in the developing limb buds as well their progenies in adults.^40^ *Prrx1*Cre mice have been successfully used to target mesenchymal progenitor cells in adult bone marrow^51^ and mesenchymal progenitor cell-derived chondroprogenitors, osteoprogenitors in bones, ligaments, tendons^52^ and adipocytes,^53^ all of which are of mesenchymal origin.^54^ Therefore, the mesenchymal specificity of the *Prrx1*Cre strain is well established. Herein, we show that in skeletal muscle, *Prrx1*Cre exclusively labels mesenchymal cells with the typical FAP phenotype (expressing CD34, Sca-1 and PDGFRα antigens at the cell surface but not ITGA7, which is specific to SCs in the muscle^25^) both before and after muscle injury. Furthermore, these cells are reticulated and scattered along myofibers and do not form myofibers in the regenerating muscle, consistent with the known function of FAPs in coordinating muscle repair rather than forming new myocytes.^25–29^ Therefore, although *Prrx1*Cre targets many other mesenchymal cells in various tissues of the body, it exclusively labels FAPs in the noninjured skeletal muscle. From these considerations, we conclude that our lineage-tracing models establish that the cells of origin that form NHOs following SCI are mesenchymal progenitor cells, not muscle satellite cells.

Other studies using models of FOP,^32,34^ calcific tendinitis and HO subsequent to tenectomy^43^ suggest that circulating mesenchymal progenitors contribute to HO formation. Therefore, we explored whether *Prrx1*-expressing cells circulate following SCI or muscle injury using *Prrx1*^ZsG^ mice. As parabiosis experiments are banned in Australia for ethical reasons, we instead tracked these cells by flow cytometry of the peripheral blood. No cells expressing high levels of ZsGreen or with a FAP phenotype were detected in the blood of the *Prrx1*^ZsG^ mice at multiple timepoints following SCI and muscle injury, whereas these ZsGreen^high^ cells were very abundant in both naïve and injured muscles. Therefore, considering the high abundance of *Prrx1*-expressing FAPs in the muscle itself and their extreme rarity in the blood, it is very unlikely that NHOs following SCI are derived from circulating mesenchymal cells. Although our SCI-induced NHO model does not involve BMP signaling and endochondral ossification, our result is consistent with a report showing that in a model of HO induced by implantation of BMP-2 Matrigel, HOs are not derived from circulating mesenchymal progenitors but from local FAPs.^35^

As lineage tracing experiments are not possible in humans, we attempted to validate our results from mice with NHO biopsies resected from patients with SCI and traumatic brain injury. We isolated progenitor cell populations from skeletal muscle residues surrounding these resected NHOs. Because of the small amount of muscle tissue on the surgical residues, adherent cells were amplified before and after sorting, possibly resulting in slight phenotypic changes. We sorted CD56^+^ cells known as myogenic progenitor cells.^48^ Although they expressed CD90, CD105 and CD73 in culture, they were negative for PDGFRα and expressed the key myogenic regulatory transcription factors *MYF5* and *MYOD1*. Conversely, PDGFRα^+^ cells had the classical mesenchymal cell phenotype, were CD56^−^ and did not express the myogenic regulatory factors *MYF5* or *MYOD1*. Therefore, human muscle CD56^+^ and PDGFRα^+^ populations correspond to *Pax7*- and *Prrx1*-expressing SCs and FAPs in mice. In our in vitro osteogenic assay, PDGFRα^+^ cells always had a higher osteogenic potential than CD56^+^ muscle progenitors, especially in the presence of the proinflammatory cytokine OSM. PDGFRα^+^ cells were also more efficient in developing ectopic bones with human-derived osteocytes and osteoblasts and hematopoietic marrow when subcutaneously implanted in a conductive biomaterial in immunodeficient mice. Our findings are consistent with a previous report that showed that although both populations displayed osteogenic potential in vitro, PDGFRα^+^ cells had a far superior ability to form ectopic bones when transplanted on a supportive scaffold into immunodeficient mice, as previously reported.^55^ Taken together, these results strongly support the mesenchymal and nonmyogenic origin of NHOs in patients with SCI.

Together, our lineage-tracing experiments suggest that NHO development subsequent to SCI is a pathology of the injured muscle, where muscle regeneration is deregulated with failure of muscle SCs to regenerate myocytes. We found that after SCI, fewer FAPs underwent apoptosis at day 3 following muscle injury, while FAPs continued to proliferate and incorporate BrdU even at days 12-14 post-injury. This finding suggests that subsequent to SCI, extensive and uncontrolled FAP survival, proliferation, and differentiation results in the formation of extensive fibrotic areas in which osteogenic differentiation leads to heterotopic bone formation rather than muscle repair. This finding suggests that severe CNS trauma, such as SCI, can reprogram FAPs in injured muscle, whereby the apoptotic process that occurs in proliferative FAPs to reduce their numbers back to baseline levels after muscle injury^26,27,29^ is impaired in the context of SCI. As a consequence, proliferating FAPs fail to undergo apoptosis and continue to accumulate in the injured muscle, which becomes fibrotic, followed by osteogenic differentiation of these FAPs into osteoblast-like cells, leading to the development of heterotopic bone tissue within injured muscles. From this perspective, we showed that SCI causes the selective upregulation of PDGFRα expression at the surface of muscle FAPs independent of the muscle injury itself, with no such effect on SCs. This finding is consistent with the observation that sciatic nerve transection also upregulates PDGFRα expression in denervated hindlimb muscle,^28^ resulting in FAP accumulation and muscle fibrosis.^28,56,57^ While heterotopic ossifications were not reported in these CDTX-injured denervated hindlimbs,^56,57^ we have shown that in the presence of an SCI, denervation further increases NHO volumes.^58^ Therefore, our data suggest that SCI reprograms FAPs in skeletal muscles, resulting in increased PDGFRα expression, reduced apoptosis and persistent proliferation in injured muscles. As PDGF and its receptors are prime survival and proliferative signals in mesenchymal cells, this may contribute to NHO development. This conclusion will need to be further confirmed in future work, and it will be of interest to identify which factors (neural or systemic) cause upregulation of PDGFRα expression on FAPs in response to SCI.

In conclusion, our lineage tracing experiments in mice and xenotransplantation of human muscle cells associated with NHOs reveal that NHO is a pathology of the injured muscle in which SCI reprograms FAPs in the damaged muscle with fibrotic hyperproliferation and osteogenic differentiation instead of apoptosis. Importantly, there was no transdifferentiation of muscle satellite cells. These findings clarify the question of the cells of origin of NHOs, demonstrating for the first time that severe CNS traumas can reprogram FAPs in skeletal muscles. This new knowledge may provide new therapeutic opportunities to reduce NHO development in victims of severe CNS traumas.

## Materials and methods

### Animal ethics and sources

All experimental procedures in mice were approved by the Health Sciences Animal Ethics Committee of The University of Queensland and followed the Australian Code of Practice for the Care and Use of Animals for Scientific Purposes. In vivo osteogenic assays in nude mice were approved by the French Institutional Animal Care and Use Committee CAPSUD/N°26 under ethics approval #9516/2017040715214163. C57BL/6 mice were obtained from the Animal Resource Center (Perth, Australia).

### Lineage tracing animal models

B6.Cg-*Pax7*^*tm1(Cre/ERT2)Gaka*^/J, B6.Cg-Tg(*Prrx1-cre*)1Cjt/J and B6.Cg-*Gt(ROSA)26Sor*^*tm6(CAG-ZsGreen1)Hze*^/J mice were purchased from the Jackson Laboratory. The B6.Cg-*Gt(ROSA)26Sor*^*tm6(CAG-ZsGreen1)Hze*^/J reporter strain (or *ROSA26*-LoxP-STOP-loxP-ZsGreen) has a CAG promoter-driven floxed STOP codon cassette ZsGreen cassette knocked into the *ROSA26* gene trap locus; therefore, ZsGreen is only expressed once recombined by Cre recombinase. To specifically trace SCs, we selected B6.Cg-*Pax7*^tm1(Cre/ERT2)Gaka^/J (in brief *Pax7*-CreERT2) mice in which Cre recombinase is fused with a mutant estrogen receptor (CreERT2) fusion protein sequence that makes it tamoxifen-inducible and knocked-in together with an intraribosomal entry site downstream of the stop codon of the *Pax7* gene, which is specifically expressed in SCs.^39^ *Pax7*^ZsG^ mice were generated by crossing *Pax7*-CreERT2 with *ROSA26*-LoxP-STOP-loxP-ZsGreen mice (Fig. 1a). The primers and PCR conditions for genotyping are detailed in Table S2. CreERT2 nuclear translocation and subsequent ZsGreen expression by excision of the floxed STOP cassette in *Pax7*^ZsG^ mice was induced by daily oral gavage with tamoxifen 125 mg·kg^−1^ for 4 days followed by a 2-week rest prior to surgery or CDTX intramuscular injection. To trace FAPs, we selected B6.Cg-Tg(*Prrx1-cre*)1Cjt/J transgenic mice (in brief *Prrx1*-Cre), in which Cre recombinase expression is controlled by the *Prrx1* promoter/enhancer specifically expressed in mesenchymal stem and progenitor cells. *Prrx1*^ZsG^ mice were generated by crossing *Prrx1*-Cre males with *ROSA26*-LoxP-STOP-loxP-ZsGreen female mice. The genotype of *Prrx1*-ZsGreen mice was confirmed by PCR as recommended by the Jackson Laboratory.

### Mouse model of SCI-induced NHOs

For induced NHO formation, 5- to 8-week-old female mice underwent spinal cord transaction surgery (SCI) at T11-13 or control sham surgery (surgical incisions were only created on skin and muscle without damaging the spinal cord) followed by an intramuscular injection (i.m.) of 0.32 mg·kg^−1^ purified *Naja pallida* CDTX (Latoxan, France) or an equal volume of sterile phosphate buffered saline (PBS).^20^ Mice were euthanized by CO_2_ asphyxiation at the indicated time points. Hamstring muscle samples were processed for flow cytometry or histological analysis as stated below.

### Flow cytometry

Single cells from hamstring muscles were isolated using a skeletal muscle dissociation kit (Miltenyi Biotech) and GentleMACS Dissociator tissue homogenizer (Miltenyi Biotec, Macquarie Park, Australia) as previously described.^20^ Blood samples were collected by terminal cardiac puncture 1, 2, 3 and 7 days post-surgery in heparinized tubes under anesthesia (2%–3% isoflurane). Red blood cells were lysed in 150 mmol·L^−1^ NH_4_Cl, 10 mmol·L^−1^ NaHCO_3_, and 1 mmol·L^−1^ EDTA, pH = 7.4 buffer, with mixing, and the remaining cells were washed again in PBS containing 10% newborn calf serum (NCS) and 2 mmol·L^−1^ EDTA prior to staining.

For characterization of ZsGreen^+^ SC-derived cells and ZsGreen^+^ FAP-derived cells, single cell suspensions were stained with monoclonal antibodies for surface markers: biotinylated anti-lineage (CD3ε, CD11b, B220, Gr1, Ter119) and streptavidin-APCCy7, CD45-BV785, CD31-BV421, CD34-e660, anti-Sca1-PECy7, anti-PDGFRα-BB700, anti-ITGA7-PE and Fixable Viability Stain 700 (FVS700). For PDGFRα expression analysis in C57BL/6 mice, cells were stained for the surface markers with anti-Ter119-FITC, CD45-BV785, CD31-BV421, CD34-e660, anti-Sca1-PECy7, anti-PDGFRα-PE and FVS700. For mesenchymal stem cell mobilization, cells were stained with anti-lineage (CD3, B220, Ter119)-PerCpCy5.5, CD45-BV785, CD11b-BV510, F4/80-PE, CD31-BV421, CD34-e660, and Sca1-PECy7 (antibodies detailed in Table S3). Flow cytometry analysis was performed using a CytoFlex flow cytometer equipped with 405 nm, 488 nm, 561 nm and 640 nm solid-state lasers (Beckman Coulter) or alternatively with a CyAn flow cytometer equipped with 405 nm, 488 nm and 640 nm solid-state lasers. Data analysis was performed using FlowJo v10.6.1 software following compensation with single color controls.

### MicroCT analyses

NHO volumes were measured in vivo in the Inveon PET-CT multimodality system (Siemens Medical Solutions, Inc.). Mice were anesthetized with a 2% isoflurane oxygen mixture. The parameters used were as follows: 360° rotation, 180 projections, 80 kV voltage, 500 μA current, and effective pixel size 36 μm. After 3D image reconstruction, NHO volume was quantified in the Inveon Research Workplace (Siemens Medical Solutions, Inc.) as previously described.^20,42^

### Histological analyses of mouse muscles

Dissected muscle was fixed in 4% paraformaldehyde for 24 h followed by decalcification in 14% EDTA for 4 days. The solution was replaced with 10%, 20% and 30% sucrose/PBS every 24 h. Muscles were mounted in optimum cutting temperature compound (OCT), frozen in isopentane in liquid nitrogen and stored at -80 °C. Muscle samples were sectioned at 8 µm using a Leica Cryostat CM1950. Every 8^th^ section was stained with hematoxylin & eosin and examined for the presence of heterotopic ossification (HO). Slides consecutive to those with confirmed HO were subsequently subjected to immunofluorescence staining. After antigen retrieval with mild Proteinase K digestion (50 μg·mL^−1^ Proteinase K (Ambion) for 2 min), the sections were incubated for 10 min in PBS-Triton 0.5% and subsequently blocked for 1 h with 10% FBS/10% NGS in 0.1% Tween 20 Tris-HCl buffered saline (TBS-Tween 0.1%). The sections were incubated with primary antibodies (collagen type I (US Biological C7510-13), osteocalcin (EnzoLife Sciences ALX-210-333) or rabbit IgG control (Thermo Fisher 31235) diluted to 1 μg·mL^−1^ in TBS-Tween 0.1% for 1 h at room temperature. The sections were washed with TBS-Tween 0.1%, incubated with biotin-labeled goat-anti-rabbit IgG secondary antibody (Vector Labs) for 20 min, washed and incubated with Alexa Fluor 647-labeled streptavidin (Thermo Fisher) for 20 min. After counterstaining with 6-diamidino-2-phenylindole (DAPI) at 0.5 μg·mL^−1^ in TBS-Tween 0.1%, the sections were mounted with ProLong™ Gold Antifade Mountant mounting medium (Thermo Fisher). Images of histological stains were captured at 20X magnification using an Olympus VS120 Slide scanner Microscope. Fluorescent images were captured on a Perkin Elmer Vectra III Spectral Scanner Microscope and spectrally unmixed using InForm software (Perkin Elmer) or an Olympus BX63 Upright Epifluorescence microscope. The number of osteocalcin^+^ NHO nodules was counted in both *Pax7*^ZsG^ (n = 4) and *Prrx1*^ZsG^ (n = 3) mice. Two sections from the same mice (>120 µm apart) were stained, quantified and recorded in relation to the presence or absence of ZsGreen^+^ cells.

### Inhibition of BMP-2 signaling by LDN-193189 in vitro

Bone marrow mesenchymal cells^21^ were seeded in a 96-well plate (1.5×10^3^ cells per well) and maintained in αMEM + 20% FCS + 1% PSG until reaching confluence. The medium was then replaced with osteogenic medium [αMEM + 20% FCS + 1% PSG containing β-glycerophosphate (10 mmol·L^−1^), phospho-ascorbic acid (200 μmol·L^−1^), CaCl_2_ (2 mmol·L^−1^) and dexamethasone (0.2 μmol·L^−1^)] and recombinant human BMP-2 (100 ng·mL^−1^) with additional LDN-193189 at 10, 100, 1 000 nmol·L^−1^ or an equivalent volume of dimethyl sulfoxide vehicle. The medium was refreshed every 3 days. Mineralization on day 7 was quantified using Alizarin Red staining.^21^ In brief, cells were washed with PBS and fixed in 4% paraformaldehyde for 30 min. After removal of the fixative, the cells were air-dried overnight, stained with 1% Alizarin Red S solution at RT on a shaker for 5-10 min, washed with PBS and air-dried overnight. Staining was dissolved in 10% cetylpyridinium chloride (CPC) containing sodium phosphate (10 mmol·L^−1^) pH 7.0 at RT on a shaker for 15 min and transferred to a new 96-well plate to measure observance at 562 nm using a Thermo Fisher Multiskan reader.

### Mouse muscle RNA extraction and qRT-PCR

Female C57BL/6 mice underwent SCI or sham surgery followed by i.m. injections of CDTX or PBS. Four days after surgery, hamstring muscle samples were harvested and snap frozen in liquid nitrogen. Frozen muscle was homogenized in TRIzol with an IKA T10 basic homogenizer on ice for 30 seconds 3 times with 10 second intervals followed by incubation at RT for 30 min. Tissue debris was removed after centrifugation at 12 000 × *g*, and the supernatant was transferred to fresh tubes and mixed with a 1/5 volume of chloroform. After centrifugation at 12 000 × *g* and 4 °C for 15 min, the aqueous phase was transferred to a fresh tube, and RNA was precipitated by isopropanol, washed in 75% ethanol, air-dried and dissolved in RNase/DNase-free water. RNA was quantified using a Nanodrop system (Thermo Scientific). cDNA was synthesized using a SeniFast cDNA synthesis kit following the manufacturer’s instructions. mRNA expression was analyzed using a single-step reverse transcription quantitative real-time polymerase chain reaction (RT-qPCR) TaqMan system. The reaction was prepared following the instructions of TaqMan™ fast PCR Master Mix and *Bmp2, Bmp4, Bmp7* and *Rps20* assays (Table S4). Reactions were read using an Applied Biosystems Viia7 Real-time PCR system. Ct values were normalized by the expression of the housekeeping gene *Rps20* and are presented as ratios to that of housekeeping genes.

### In vivo LDN-193189 treatments

C57BL/6 mice underwent SCI and CDTX intramuscular injection as indicated above on day 0. Mice were treated with vehicle (6% DMSO in PBS) or LDN-193189 (30 mg·kg^−1^) via intraperitoneal (i.p.) injection twice daily from days 0-14 or days 14-21, which is effective in reducing HO volumes in a mouse model of FOP driven by the *ACVR1*^Q207D^ missense mutation.^44^ Animals were randomly allocated to the vehicle or treatment groups after surgery (while the animals were still under anesthesia) but prior to treatment commencements. Therefore, grouping was not affected by recovery from surgery.

### In vivo FAP apoptosis measurement

Mice underwent SCI or sham surgeries and CDTX injection and, samples were harvested 3 days post-surgery. Muscle cells were isolated as described above followed by surface marker staining: biotinylated anti-lineage (CD3ε, CD5, B220, CD11b, Gr1, Ter119), CD45-BV785, CD31-BV421, CD34-e660, Sca1-PECy7 and anti-ITGA7-PE antibodies followed by streptavidin (SAV)-BUV395. After surface marker staining, the cells were washed in 1X PBS and then binding buffer (10 mmol·L^−1^ HEPES pH=7.4, 150 mmol·L^−1^ NaCl, 5 mmol·L^−1^ MgCl_2_, 5 mmol·L^−1^ KCl, 1.8 mmol·L^−1^ CaCl_2_). Next, the cells were stained with Annexin V-FITC (1/50 dilution) in annexin binding buffer at RT for 20 min. After excess Annexin V was washed away, the cells were resuspended in Annexin binding buffer, and a 1/5 volume of 7AAD was added to the kit. Flow cytometry analysis was performed using a BD LSR Fortessa X20 cytometer equipped with 355 nm, 405 nm, 488 nm, 561 nm and 640 nm solid-state lasers.

### In vivo FAP proliferation measurement by BrdU incorporation

For measurement of in vivo FAP proliferation during NHO development, mice underwent SCI or sham surgeries and CDTX injection. Two days prior to tissue harvest at day 14 post-surgery, the mice were given drinking water containing 0.5 mg·mL^−1^ BrdU (Sigma-Aldrich cat# B5002) together with twice daily i.p. injection of BrdU for the last 2 days before tissue sampling. One mouse from the SCI + CDTX group and one mouse from the sham+CDTX group were not treated with BrdU and used as a negative control for BrdU staining.

Muscle cells were isolated as described above followed by surface marker staining: anti-lineage (CD3ε, CD5, B220, CD11b, Gr1, Ter119)-Pacific blue, CD45-BV785, CD31-PE, CD34-e660, and anti-Sca1-PECy7. The cells were then fixed and permeabilized using a BrdU flow kit (BD Pharmingen, cat# 559619) following the manufacturer’s instructions. The fixed cells were then treated with DNase I (13 μg per 1–2 ×10^6^ cells) in DNase reaction buffer (10 mmol·L^−1^ Tris-HCl, 0.5 mmol·L^−1^ CaCl_2,_ 2.5 mmol·L^−1^ MgCl_2_ in PBS, pH 7.4) at 37 °C for 1 h. BrdU incorporation in DNA was labeled with anti-BrdU-FITC monoclonal antibody in buffer containing 10 μg·mL^−1^ blocking mouse IgG at RT for 1 h. The cells were then washed and analyzed by flow analysis using a BD LSR Fortessa X20 cytometer.

### Isolation of human BM-MSCs, PDGFRα^+^ and CD56^+^ cells

All NHO and muscle samples were obtained with the informed consent of the patients and approval from the People’s Protection Committee (CPP approval n°09025) and from the National Commission for Informatics and Liberties (CNIL approval n° Eyo1066211J). NHO biopsies were taken from the hips of 12 patients whose characteristics are provided in Table 1.

**Table 1:** Patient information

Patient #	Sex	Age/year	Trauma	NHO location
1	M	33	SCI	Hip
2	F	28	TBI	
3	M	35	TBI	
4	M	26	SCI	
5	M	35	TBI	
6	M	52	TBI	
7	M	65	SCI	
8	F	72	Stroke	
9	F	47	Stroke	
10	M	48	TBI	
11	F	63	TBI	
12	F	71	TBI	

*M* male, *F* female, *SCI* spinal cord injury, *TBI* traumatic brain injury

Muscle surrounding NHOs was collected from NHO surgical waste following their excision from patients with brain injuries, spinal cord injuries or strokes. NHO resection surgeries were performed at Raymond Poincaré Hospital (Garches, France). Muscle fragments were minced using a scalpel and small scissors, placed in a 50 mL Falcon tube and incubated in 1.5 mg·mL^−1^ pronase (#10165921001 Sigma-Aldrich) in α-MEM for 45 min in a 37 °C water bath. After the addition of α-MEM supplemented with 15% FCS and 1% antibiotics, the cell suspension was filtered through a 100 µm cell strainer followed by a 40 µm cell strainer (BD Falcon). Isolated muscle progenitor cells (MPCs) were maintained in α-MEM supplemented with 15% FCS, 1% antibiotics and 10 ng·mL^−1^ basic fibroblast growth factor (bFGF) (R&D Systems). Human MPCs were trypsinized and incubated for 30 min with biotinylated anti-human PDGFRα (R&D system) and CD56-PE (clone B159, BD Pharmingen) monoclonal antibodies in PBS with 2% FCS and 2 mmol·L^−1^ EDTA. The cells were washed and incubated for 30 min with streptavidin-APC/Cy7 and the viability dye 7-AAD (Sony). The cells were washed and filtered through a 30 µm cell strainer (Sysmex) and sorted using a FACSAria III SORP sorter (BD Biosciences). PDGFRα^+^ and CD56^+^ cells were seeded at 3 000 per cm^2^ in α-MEM supplemented with 20% FCS, 1% antibiotics and 10 ng·mL^−1^ bFGF (R&D Systems).

BM-MSCs were isolated by plastic adhesion and expanded to 70%–80% confluence in α-MEM supplemented with 10% FCS and 1% antibiotics. Cells were subsequently seeded at 4 000 per cm^2^.

### Flow cytometry phenotyping of human PDGFRα^+^ and CD56^+^ cells

PDGFRα^+^ and CD56^+^ cell suspensions (passages 4 and 5) were stained with monoclonal antibodies against the surface markers CD31-PE, CD34-APC, CD45-APC, CD73-PE, CD90-APC, and CD105-PE (Table S3). Flow cytometry analysis was performed using a CytoFlex flow cytometer (Beckman Coulter). Data analysis was performed using FlowJo v10.6.1 software following compensation with single color controls.

### qRT-PCR of human PDGFRα^+^ and CD56^+^ cells

PDGFRα^+^ and CD56^+^ cells (passages 3 to 5) were lysed in QIAzol (Qiagen) followed by chloroform/isopropanol total RNA extraction. Reverse transcription was performed using a Reverse Transcriptase Core Kit (Eurogentec). qRT-PCR was performed on a LightCycler 480 instrument (Roche) using a QuantiTect SYBR Green RT-PCR Kit and QuantiTect Primers (Qiagen). Three housekeeping gene mRNAs (*GAPDH*, *PPIA*, *HPRT*) were selected based on their expression stability using geNorm analysis. *MYF5* and *MYOD1* quantification was performed as the geometric mean of the quantifications obtained with each reference RNA.

### In vitro osteogenic differentiation and quantification of mineralization of human cells

PDGFRα^+^ and CD56^+^ cells were seeded in 24-well plates at 3 000 per cm^2^ in α-MEM supplemented with 10% FCS and 1% antibiotics. Once the cells adhered to the wells, the medium was replaced by α-MEM supplemented with 10% FCS, 1% antibiotics and 0.052 μg·mL^−1^ dexamethasone, 12.8 μg·mL^−1^ ascorbic acid, and 2.15 mg·mL^−1^ ß-glycerophosphate (Sigma-Aldrich) to induce osteogenic differentiation. Human recombinant OSM was added at 100 ng·mL^−1^ (Miltenyi Biotec). The cells were cultured for 14 days at 37 °C in a 5% CO_2_ atmosphere, and the medium was changed twice a week. Quantification of mineralization was performed using Alizarin Red S staining. The cells were washed with PBS, fixed in 70% ethanol, quickly washed with distilled water and incubated for 5 min in 20 g·L^−1^ Alizarin Red S, pH = 4.2 (Sigma-Aldrich). The cells were washed with distilled water and dried. Alizarin Red S dye was extracted with 0.5 mol·L^−1^ hydrochloric acid and 5% SDS and quantified by spectrophotometry at 405 nm.

### In vivo osteogenic assays with human cells

Subconfluent human BM-MSCs and PDGFRα^+^ and CD56^+^ cells (2–3 amplification passages) were collected for subcutaneous implantation into the flanks of 10-week-old male nude mice (Janvier Labs). Implants were prepared by mixing 2 × 10^6^ cells with sterile 80–200 µm particles (60% hydroxyapatite/40% ß-tricalcium phosphate) (Graftys) and 100 μL of human plasma in a 1 mL syringe as described.^59^ Control “plasma implants” did not contain cells. Ten microliters of 2% CaCl_2_ in water was added to induce coagulation. Fifteen weeks after implantation, animals were euthanized, scaffolds were collected and fixed in 4% paraformaldehyde overnight at 4 °C. The following day, scaffolds were washed with PBS and decalcified in 20% EDTA pH = 8 solution for 48 h. Implants were washed with PBS and dehydrated with a graded series of ethanol solutions prior to paraffin embedding (Thermo Scientific).

### Implant histology and immunohistochemistry

Paraffin sections (4 μm) were dried, deparaffinized, and stained with hematoxylin eosin safranin (HES) (Dako). For immunohistochemical staining, 4 µm paraffin sections were incubated with antigen retrieval citrate-based solution pH = 9 (Vector Laboratories) at 95 °C for 15 min. The sections were incubated in Bloxall blocking solution (Vector Laboratories) for 10 min to inactivate endogenous peroxidase. FC receptor blocking reagent (Innovex Biosciences) was then added for 45 min. All sections were incubated in PBS-10% FCS for 30 min with either anti-lamin A/C antibody (dilution 1/200, clone EPR4100, Abcam) or anti-osterix/SP7 antibody (dilution 1/100, Abcam) diluted in PBS-5% FCS-1.5% BSA overnight incubation at 4 °C. The sections were washed with PBS-0.1% Triton and incubated in ImPRESS Reagent anti-rabbit IgG (Vector Laboratories) for 30 min. Then, the sections were washed with PBS-Triton 0.1% and incubated in HistoGreen substrate solution (Linaris) for 1 min. Counter coloration was performed using Fast Nuclear Red (Vector Laboratories). All sections were analyzed using a Pannoramic Midi II slide scanner and Case Viewer software (3D HISTECH, Ltd.).

### Statistics

Statistical differences were calculated using the Mann-Whitney test or one-way ANOVA with Tukey’s multiple comparison test with Prism v7 or 8 software (GraphPad software, La Jolla, CA).51. The data visualization in Fig. 4 was also performed using the Rstudio v1.1456, tidyverse and ggplot2 packages.

## Supplementary information

Supplementary figures S1-S6

Supplementary tables S1-S5
