# Supplementary material for: Spinal cord injury reprograms muscle fibroadipogenic progenitors to form heterotopic bones within muscles
Source: Bone Res. 2022 Feb 25;10:22. doi: 10.1038/s41413-022-00188-y (PMC8881504; doi:10.1038/s41413-022-00188-y)
Supplement: Supplementary file 2 — Supplementary tables S1-S5 [file 41413_2022_188_MOESM2_ESM.pdf]

Supplementary Table 1. Percentage of phenotypic SCs, FAPs and endothelial cells in non-hematopoietic cells from the hamstring muscles of *Pax7*^{ZsG} and *Prrx1*^{ZsG} mice at 14 days post PBS or CDTX injection measured by flow cytometry.

Mouse strains	Intramuscular injections	Satellite cells	Fibro-adipogenic progenitors	Endothelial cells
Pax7^{ZsG}	PBS	11.0 ± 2.6	15.7 ± 5.1	0.56 ± 0.12
Prrx1^{ZsG}	PBS	11.1 ± 2.3	11.8 ± 1.8	0.66 ± 0.19
Pax7^{ZsG}	CDTX	9.9 ± 0.5	16.72 ± 7.3	0.72 ± 0.33
Prrx1^{ZsG}	CDTX	9.8 ± 0.9	15.2 ± 3.4	0.81 ± 0.23

Data are presented as mean ± SD (n=4 mice/group for *Pax7*^{ZsG} and n=3 mice/group for *Prrx1*^{ZsG}). There was no significant difference between the different strains as determined by one-way ANOVA.

Supplementary Table 2. Genotyping PCR reagents and conditions

Genes	Primers	Primer sequences	PCR Conditions
Pax7-CreERT2	Pax7 common F	5'GCTGCTGTTGATTACCTGGC	94 °C 15s, 60°C 30s, 72°C 60s 30 cycles
	Pax7 wt R	5'CTGCACTGAGACAGGACCG	
	Pax7 Mutant R	5'CAAAAGACGGCAATATGGTG	
Rosa26R-Zsgreen	Rosa26 wt F	5' AAG GGA GCT GCA GTG GAG TA	94 °C 15s, 62°C 30s, 72°C 60s 35 cycles
	Rosa26 wt R	5' CCG AAA ATC TGT GGG AAG TC	
	Rosa26-Zsgreen Mut R	5' GGC ATT AAA GCA GCG TAT CC	
	Rosa26-Zsgreen Mut F	5' AAC CAG AAG TGG CAC CTG AC	
Prrx1Cre	Cre forward	5' GAGTGATGAGGTTCGCAAGA	94 °C 15s, 58°C 30s, 72°C 60s 35 cycles
	Cre reverse	5' CTACACCAGAGACGGAAATC	

Supplementary Table 3. Antibodies used in the study.

1) **Flowcytometry (anti-mouse)**

Anti-mouse	Catalogue	Company	Clone	Dilution
B220- Biotin	103204	Biolegend	RA3-6B2	1:200
CD3-Biotin	100304	Biolegend	145-2C11	1:200
CD11b- Biotin	101204	Biolegend	M1/70	1:200
CD31-BV421	102423	Biolegend	390	1:200
CD34-e660	50-0341-82	eBioscience	RAM34	1:75
CD45-BV785	103149	Biolegend	30-F11	1:200
F4/80-PE	123110	Biolegend	BM8	1:500
GR1- Biotin	108404	Biolegend	RB6-8C5	1:200
α7integrin-PE	53-0010-05	Ablab	R2F2	1:3000
PDGFRα (CD140a)-BB700	742176	BD Bioscience	APA5	1:200
PDGFRα (CD140a)-PE	135905	Biolegend	APA5	1:100
Sca1-PECy7	108114	Biolegend	D7	1:300
Ter119- Biotin	116204	Biolegend	TER-119	1:200
Ter119-FITC	116206	Biolegend	TER-119	1:300
Fixable Viability Stain 700	564997	BD Bioscience	-	1:15000
SAV-APCCy7	554063	BD Bioscience	-	1:200
B220-PercpCy5.5	103236	Biolegend	RA3-0B2	1:200
Ter119-PercpCy5.5	116228	Biolegend	TER-119	1:200
CD3-PercpCy5.5	100328	Biolegend	145-2C11	1:200
Ly6G-APCCy7	127624	Biolegend	1A8	1:200
CD11b-BV510	101263	Biolegend	M1/70	1:200
CD3-Pacific blue	100334	Biolegend	145-2C11	1:200
GR1-Pacific blue	108430	Biolegend	RB6-8C5	1:200
B220-Pacific blue	103227	Biolegend	RA36B2	1:200
CD11b-Pacific blue	101224	Biolegend	M1/70	1:200
Ter119-Pacific blue	116231	Biolegend	TER-119	1:200
CD5-Pacific blue	100642	Biolegend	53-7.3	1:200

Anti-mouse	Catalogue	Company	Clone	Dilution
CD31-PE	102407	Biolegend	390	1:200
FITC BrdU Flow Kit	559619	BD Biosciences		1:20
Annexin V-FITC	640906	Biolegend		1:50

2) Flowcytometry (anti-Human)

Antibody	Catalogue	Company	Clone	dilution
huCD31-PE	555446	BD Biosciences	WM59	1:10
huCD34-APC	IM2472	Beckman Coulter	581	1:10
huCD45-APC	IM2473	Beckman Coulter	J33	1:10
huCD73-PE	550257	BD Biosciences	AD2	1:10
huCD90-APC	559869	BD Biosciences	5E10	1:10
huCD105-PE	PN A07414	Beckman Coulter	1G2	1:10
huCD56-PE	555516	BD Biosciences	B159	1:10
huPDGFRα-Biotin	BAF1322	R&D Systems	-	1:10
Streptavidin APC/Cy7	2626040	Sony	-	1:200
7-AAD (7-Aminoactinomycin D)	A1310	Molecular probes	-	
huLamin A/C	ab108595	Abcam	EPR4100	1:100
hu-muOsterix/SP7	ab22552	Abcam	-	1:100

3) Immunofluorescence staining

Antibody	Catalogue	Company	Concentration
Collagen Type I	C7510-13	US Biological	1 ug/ml
Osteocalcin	ALX-210-333	EnzoLife Sciences	1ug/ml
Rabbit IgG control	31235	ThermoFisher	1ug/ml
biotin-labelled goat-anti-Rabbit IgG secondary antibody	BA-1000	Vector Labs	

Streptavidin, Alexa Fluor™ 647 conjugate	S21374	Invitrogen	

4) **Western blot**

Antibody	Catalogue	Company	Clone	Dilution
p-Akt (S473)	4060s	Cell Signaling		1:1000
Total Akt (c67E7)	4691	Cell Signaling		1:1000
IRDye® 800CW Donkey anti-Rabbit IgG (H + L)	LCR -926-32213	Licor		1:15000

Supplementary Table 4: qRT-PCR primer probe sets

	Catalogue	Company
SensiFast	BIO65054	Bioline
TaqMan™ Fast Advanced Master Mix	4444557	ThermoFisher
Rsp20 gene expression assay	Mm02342828_g1	ThermoFisher
Bmp2 gene expression assay	Mm01340178_m1	ThermoFisher
Bmp4 gene expression assay	Mm00432087_m1	ThermoFisher
Bmp7 gene expression assay	Mm00432102_m1	ThermoFisher

Supplementary Table 5. Other reagents.

Reagents	Company	Catalogue number
Tamoxifen	Signa-Aldrich	T5648
Cardiotoxin	Latoxan	L8102
Skeletal muscle dissociation kit	Miltenyi Biotec	130-098-305
Recombinant Mouse PDGF-BB (carrier-free)	Biolegend	RUO-558802
Recombinant human BMP2	Peprotech	120-02C-10
LDN-193189	Cayman Chemical	19396
Dasastinib	Tocris	6793
DAPI	Signa-Aldrich	D5942
ProLong™ Gold Antifade Mountant	Invitrogen	P36930
Tissue-Tek® O.C.T.™ Compound	Tissue-Tek	IA018
Superfrost Plus™ Adhesion Microscope Slides	Thermo Fisher Scientific	MENSF41296SP
Mx 35 premier + blades	Thermo Scientific	3052835
cOmplete™ ULTRA Tablets, EDTA-free, glass vials Protease Inhibitor Cocktail	Roche	05892953001
4-12% Bis-Tris pre-cast gel	Invitrogen	NW04122
Thermofisher nitrocellulose mini stacks	ThermoFisher	B23002
Odyssey Blocking Buffer	Licor	921-50000
Restore PLUS Western Blot Stripping Buffer	ThermoFisher	46430
Paraformaldehyde	Signa-Aldrich	P6148
EDTA disodium salt	Astral Scientific	BIOEB0185-500g
Alizeran red S	Signa-Aldrich	A5533
Cetylpyridinium chloride	Signa-Aldrich	C0732
β-Glycerophosphate disodium salt hydrate	Signa-Aldrich	A5533
Dexamethasone	Hospira	433449
